# Hydroxycoumarin Scopoletin Inhibits Bone Loss through Enhancing Induction of Bone Turnover Markers in a Mouse Model of Type 2 Diabetes

**DOI:** 10.3390/biomedicines9060648

**Published:** 2021-06-07

**Authors:** Eun-Jung Lee, Woojin Na, Min-Kyung Kang, Yun-Ho Kim, Dong-Yeon Kim, Hyeongjoo Oh, Soo-Il Kim, Su-Yeon Oh, Sohyun Park, Kyungho Park, Young-Hee Kang

**Affiliations:** Department of Food and Nutrition and Korean Institute of Nutrition, Hallym University, Chuncheon 24252, Korea; reydmswjd@naver.com (E.-J.L.); nsm0729@hanmail.net (W.N.); mitholy@hallym.ac.kr (M.-K.K.); royalskim@hallym.ac.kr (Y.-H.K.); ehddus3290@naver.com (D.-Y.K.); ohhyeongju@gmail.com (H.O.); ky4850@naver.com (S.-I.K.); suy0411@naver.com (S.-Y.O.); sopark@hallym.ac.kr (S.P.); Kyungho.Park@hallym.ac.kr (K.P.)

**Keywords:** bone remodeling, bone turnover, osteoblasts, osteoclasts, scopoletin, type 2 diabetes

## Abstract

Diabetes induces bone deterioration, which leads to increased risk of fracture, osteopenia, and osteoporosis. Thus, diabetes-associated bone fragility has been recognized as a diabetic complication. However, the pathophysiological effects of hyperglycemia on bone turnover remain unclear. Literature evidence demonstrates that anti-diabetic medications increase the risk of fractures in individuals with type 2 diabetes. Scopoletin is a naturally occurring hydroxycoumarin potentially exhibiting anti-inflammatory and antioxidant activities and ameliorating insulin resistance as an anti-diabetic agent. However, little is known regarding the effects of scopoletin on the impairment of bone remodeling that is caused by diabetes. The aim of this study was to identify that scopoletin was capable of inhibiting the impairment of bone remodeling and turnover in a mouse model of type 2 diabetes. Submicromolar scopoletin accelerated the formation TRAP-positive multinucleated osteoclasts (40.0 vs. 105.1%) and actin ring structures impaired by 33 mM glucose. Further, 1–20 μM scopoletin enhanced bone resorption and the induction of matrix-degrading enzymes in diabetic osteoclasts. The oral administration of 10 mg/kg scopoletin elevated serum RANKL/OPG ratio and osteocalcin level reduced in db/db mice along with an increase in BMD by ~6–14%; however, it was not effective in lowering blood glucose and hemoglobin glycation. In addition, the supplementation of scopoletin elevated the formation of trabecular bones and collagen fibers in femoral epiphysis and metaphysis with a thicker epiphyseal plate and cortical bones. Furthermore, 1–20 μM scopoletin enhanced ALP activity (4.39 vs. 7.02 nmol *p*-nitrophenyl phosphate/min/mg protein) and deposits of mineralized bone nodules in cultured osteoblasts reduced by 33 mM glucose. The treatment of diabetic osteoblasts with scopoletin stimulated the cellular induction of BMP-2 and osteopontin and Runx2 transcription. Accordingly, the administration of scopoletin protected mice from type 2 diabetes-associated bone loss through boosting bone remodeling via the robust induction of bone turnover markers of both osteoclasts and osteoblasts. These findings suggest that scopoletin could be a potential osteoprotective agent for the treatment of diabetes-associated bone loss and fractures.

## 1. Introduction

There is growing evidence that diabetes and diabetic complications are associated with an increased risk of hip fractures [1,2,3]. Microstructural changes that reduce bone quality and strength, thus increasing the risk of fractures, characterize diabetic osteopathy [2,4]. The mechanisms underlying bone fragility in diabetes are not completely understood. Nevertheless, the increased risk of fractures in people with diabetes depends on age, diabetic duration, poor glycemic control, and anti-diabetic medications [4,5,6]. The increased duration of type 1 diabetes deranges the relationship between bone strength and structure, and it decreases bone toughness [7]. The higher fracture rate is observed in patients with type 2 diabetes, despite the normal to increased bone mineral density (BMD) [5,8]. Individuals with type 1 diabetes have decreased BMD, possibly because of absolute insulin deficiency and the inability of exogenous insulin to emulate endogenous insulin secretion [9]. Insulin, which acts as an anabolic agent in bone, can preserve and enhance bone density and bone strength, presumably through direct and/or indirect effects on bone formation [10]. When considering that type 2 diabetes is a common metabolic disorder with adverse effects on bone metabolism, the effect of anti-diabetic medications on bone metabolism has received increasing attention [11]. The therapy of thiazolidinediones increases the risk of fractures in elderly patients with type 2 diabetes [6,12]. Sodium-glucose co-transporter 2 inhibitors may influence bone metabolism, possibly including increased bone turnover, disrupted bone microarchitecture, and reduced bone mineral density [11]. It has been shown that insulin use is associated with high fracture risk in patients with type 2 diabetes [13,14]. Thus, the risk of fractures should be taken into consideration prior to the initiation of treatment with anti-diabetic medications.

Among the multiple factors that are responsible for increased susceptibility to fracture, the accumulation of advanced glycation end products (AGE), a biomarker that is implicated in diabetes, is associated with increased fracture risk in type 2 diabetes, having a direct detrimental effect on bone quality [4,15,16]. The accumulation of AGE in the bone collagen fibers via covalent cross-links mechanically affects the properties of the bone tissue matrix and disturbs bone remodeling, which underlies osteoporosis [16,17]. It has been shown that bone turnover is reduced in diabetes, which suggests a negative effect of hyperglycemia on bone turnover [9,18]. Bone turnover markers, such as osteocalcin and C-terminal telopeptide of type I collagen, are generally reduced in patients with type 2 diabetes when compared with non-diabetic controls [18]. Although contrastable findings have been reported for other markers, the overall evidence points toward an association of type 2 diabetes with reduced bone turnover, presumably with an imbalance between bone resorption and bone formation [4]. The suppression of bone turnover in patients with type 2 diabetes is responsible for a higher risk of fracture in a BMD-independent manner [19]. Consistently, the impairment in bone strength in type 2 diabetes is due to impaired bone quality, which is assumedly caused by low bone turnover, as well as by elevated AGE accumulation [20]. However, the involvement of abnormal bone turnover in increased fracture risk of diabetic patients needs further studies. 

**Figure 1:**
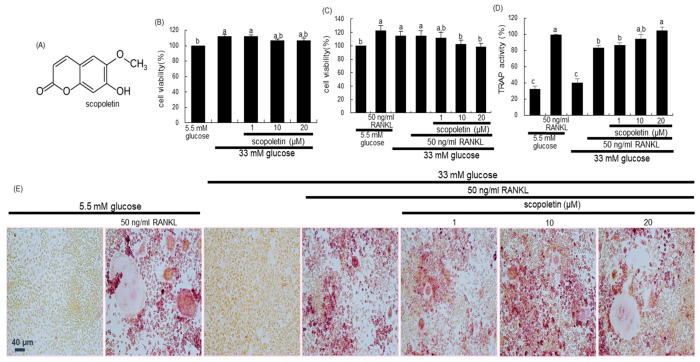
The chemical structure of scopoletin (**A**), and toxicity of pre-osteoclastic Raw 264.7 cells by glucose and scopoletin (**B**,**C**), tartrate-resistant acid phosphatase (TARP) activity (**D**), and TRAP staining (**E**). Raw 264.7 cells were cultured in α-MEM media containing 33 mM glucose for 2 days in the absence and presence of 1–20 μM scopoletin and/or 50 ng/mL receptor activator of nuclear factor-κB-ligand (RANKL). Cell viability was measured by MTT assay and expressed as percent cell survival relative to 5.5 mM glucose controls (cell viability =100%, mean ± SEM, *n* = 7). After Raw 264.7 cells were cultured for five days, the TRAP activity was measured using a kit (**D**). TRAP-positive multinucleated osteoclasts were stained and visualized under light microscopy (**E**). Scale bar = 40 μm. Values in bar graphs (mean ± SEM, *n* = 4) not sharing a common small letter indicate significant difference at *p* < 0.05.

On the basis of the literature evidence demonstrating that type 2 diabetes causes adverse effects on bone metabolism and anti-diabetic medications increase the risk of fractures, this study examined whether scopoletin enhanced bone density and strength through improving bone turnover and remodeling for type 2 diabetes. Scopoletin (Figure 1A) is a naturally-occurring hydroxycoumarin that is described as exhibiting hypotensive, xanthine oxidase-inhibitory, antioxidant, and anti-inflammatory activities [21,22,23,24]. One investigation reports that scopoletin-rich Noni leaf extract enhances bone regeneration in estrogen-deficient rats [25]. In addition, scopoletin functions as a potential anti-diabetic agent ameliorating insulin resistance [26,27]. However, little is known regarding the effects of scopoletin on the impairment of bone turnover that is caused by type 2 diabetes. Our previous study showed that the interaction of AGE and receptor for AGE was responsible for the impaired activation of diabetic osteoblasts and osteoclasts, which coumarin ameliorated [28]. The current study investigated whether scopoletin accelerated bone turnover and boosted bone remodeling in db/db mice, a mouse model of type 2 diabetes and leptin deficiency. In this study, the differentiation and induction of bone turnover markers were examined in glucose-loaded osteoclasts and osteoblasts. Scopoletin influenced the receptor activator of the nuclear factor (NF)-κB (RANK)-RANK ligand (RANKL)-osteoprotegerin (OPG) system critical for osteoclast activation and the BMP-2-Runx2 pathway that is involved in the bone formation of osteoblasts, ultimately boosting bone turnover and remodeling.

## 2. Materials and Methods

### 2.1. Chemicals

Dulbecco’s modified eagle’s media (DMEM), minimum essential medium alpha medium (α-MEM), D-glucose, and Alizarin red S dye were supplied by Sigma-Aldrich Chemical (St. Louis, MO, USA), as were all other reagents, unless specifically stated otherwise. Fetal bovine serum (FBS), trypsin–EDTA, and penicillin–streptomycin were obtained from BioWhittaker (San Diego, CA, USA). 3-(4, 5-Dimetylthiazol-yl)-diphenyl tetrazolium bromide (MTT) was purchased from DUCHEFA Biochemie (Haarlem, Netherlands). The recombinant murine sRANKL was purchased from PeproTech (Rocky Hill, NJ, USA). Scopoletin (Sigma-Aldrich Chemical, St. Louis, MO, USA) was dissolved in dimethyl sulfoxide (DMSO) for live culture with cells; the final culture concentration of DMSO was <0.5%.

### 2.2. Culture of Raw 264.7 Cells 

The murine macrophage Raw 264.7 cell line (ATCC TIB-71) was obtained from the American Type Culture Collection (Manassas, VA, USA) and then cultured in DMEM supplemented with 10% FBS, 100 U/mL penicillin, and 100 μg/mL streptomycin at 37 °C with 5% CO_2_ in air. In order to differentiate macrophages into osteoclasts, Raw 264.7 cells were seeded on a 24-well plate at the density of 1 × 10^4^ cells/mL and incubated in α-MEM (5.5 mM or 33 mM glucose) with 50 ng/mL RANKL in the absence or presence of 1–20 μM scopoletin for five days. The cell media were freshly changed every two days and mature osteoclasts were observed on day 5.

The cytotoxicity of scopoletin was determined using a MTT assay. Raw 264.7 cells that were seeded at a density of 1 × 10^4^ cells/mL on a 24-well were incubated in 5.5 mM glucose- or 33 mM glucose-loaded media and then exposed to 1–20 μM scopoletin for 48 h in the absence and presence of 50 ng/mL RANKL. The cells were treated with 1 mg/mL MTT solution and then incubated at 37 °C for 3 h, which results in the formation of insoluble purple formazan product that was dissolved in 250 μL isopropanol. The optical density was measured using a microplate reader at λ = 570 nm. Scopoletin *per se* at the doses of 1–20 μM did not cause apparent cytotoxicity (the data are not shown). The current experiments employed a dose of scopoletin in the range of 1–20 μM.

### 2.3. Measurement of Tartrate-Resistant Acid Phosphatase (TRAP) Staining and Activity

Raw 264.7 macrophages were cultured for five days on chamber slides and then incubated in α-MEM containing 5.5 mM or 33 mM glucose with 50 ng/mL RANKL in the absence or presence of 1–20 μM scopoletin. The cells were fixed with 4% formaldehyde and then stained for 30 min. with a commercially available TRAP kit (Sigma-Aldrich Chemical, St. Louis, MO, USA). The TRAP-positive multinucleated osteoclasts were visualized under light microscopy. 

For the measurement of the TRAP activity, the cells were fixed with 4% formaldehyde for 10 min. Subsequently, the dried cells were incubated in 50 mM citrate buffer (50 mM citric acid and 50 mM sodium citrate (pH 4.5)) containing 5 mM 4-nitrophenylphosphate and 10 mM sodium tartrate for 1 h. The reaction was terminated by adding 0.1 N NaOH. The absorption intensity was measured using a microplate reader at λ = 405 nm.

### 2.4. Actin Ring Staining and Bone Resorption Assay 

Raw 264.7 cells on 24-well plates were fixed in 4% formaldehyde for 10 min. and then washed with pre-warmed phosphate buffered saline (PBS). Subsequently, 10 units of the fluorescent dye rhodamine phalloidin were added to cells and then incubated for 20 min. Nuclear staining was also conducted using 1 mg/mL 4′,6-diamidino-2-phenylindole (DAPI). The fluorescent images were taken with an Axiomager optical fluorescence microscope system (Carl Zeiss, Oberkochen, Germany).

The bone resorption assay was performed by using a resorption assay kit (Cosmo Bio Co., Tokyo, Japan). After culturing for five days, the cells were washed in 5% NaOCl to remove the cells. The resorbed pits on the plate were visualized under light microscopy.

### 2.5. Western Blot Analysis

Western blot analysis was conducted with whole cell lysates and culture media that were prepared from Raw 264.7 osteoclasts and MC3T3-E1 osteoblasts. Whole cell lysates and culture media were prepared in a lysis buffer containing 1 M β-glycerophosphate, 1% β-mercaptoethanol, 0.5 M NaF, 0.1 M Na_3_VO_4_, and a protease inhibitor cocktail. Cell lysates containing equal amounts of proteins and an equal volume of culture media were electrophoresed on 8–12% SDS-PAGE and transferred onto a nitrocellulose membrane. Nonspecific binding was blocked with 5% skim milk for 3 h. The membrane was incubated overnight at 4 °C with each primary antibody of cathepsin K, matrix metalloproteinase (MMP-9), BMP-2, and osteopontin being thoroughly washed in a Tris-buffered saline-Tween 20 (TBS-T) for 10 min. The membrane was then incubated for 1 h with a secondary antibody of goat anti-rabbit IgG, goat anti-mouse IgG, and rabbit anti-goat IgG conjugated to HRP. Each target protein level was determined using immobilon western chemiluminescent HRP substrate (Millipore Corporation, Billerica, MA, USA) and Agfa X-ray film (Agfa-Gevaert N.V., Mortsel, Belgium). Incubation with the mouse monoclonal β-actin antibody (Sigma-Aldrich Chemical, St. Louis, MO, USA) was also performed for comparative controls.

### 2.6. In Vivo Animal Experiments

The current study introduced adult male db/db mice (C57BLKS/J-db/db; Japan SLC, Inc., Shizuoka, Japan) and their age-matched non-diabetic db/m littermates (C57BLKS/J-m+/m+; Japan SLC, Inc., Shizuoka, Japan). In order to measure food and water intakes, mice were conventionally housed in the individual stainless-steel hanging wire-mesh cages, with food and tap water being provided *ad libitum*. Mice were kept on a 12-h light/12-h dark cycle at 23 ± 1 °C with 50 ± 10% relative humidity under specific pathogen-free conditions, fed a standard pellet laboratory chow diet (Cargill Agri Purina, Biopia, Seongnam, Korea) at the animal facility of Hallym University. All of the experiments were approved by the Committee on Animal Experimentation of Hallym University and performed in compliance with the University’s Guidelines for the Care and Use of Laboratory Animals (hallym 2019-64). No mice died and no apparent signs of exhaustion were observed during the experimental period.

This study employed db/db mice at seven weeks of age, because they begin to develop diabetes (hyperglycemia) at the age of 7–8 weeks. The animals were allowed to acclimatize for a week before beginning the experiments. The mice were divided into three subgroups (n = 7–10 for each subgroup). The first group of mice was non-diabetic db/m control mice, and the db/db mice were divided into two groups. One group of db/db mice was orally administrated 10 mg/kg scopoletin daily for 10 weeks. 

After the 10-week scopoletin supplementation, the organ weights of liver, kidney, spleen, pancreas, and heart were measured in db/m control and db/db mice. The wet weights of the liver, kidney, pancreas, and heart of diabetic mice were much higher than those of the db/m controls, while the wet spleen weight of diabetic mice was lower (Table 1). The kidney weight was reduced by supplementing 10 mg/kg scopoletin. 

Food intake, body weight, and drinking water intake were measured in mice daily during the 10-week scopoletin supplementation. Pre-weighed food was provided in a standard stainless-steel hopper. The amount of food remaining, including any on the bottom of the cages or any that was spilled on plastic sheets placed under each cage, was measured. Water intake was manually measured by weighing the residual amounts in a water bottle. The 24-h urine collection was carried out using metabolic cages.

### 2.7. Biochemical Analysis of Blood and Bone Tissues 

A blood glucose meter was used to measure fasting blood glucose from the mouse tail veins every other week (ACCU-CHEK Performa, Roche diagnostics, Mannheim, Germany). Mouse plasma was collected by centrifugation at 3000 rpm for 10 min. at 4 °C and then stored at −20 °C prior to analysis. The plasma levels of aspartate transaminase (AST) and alanine transaminase (ALT) were determined after overnight fasting by using a Kornelab 20XT (Thermo Fisher Scientific Inc., Waltham, MA, USA). Blood glycated hemoglobin (HbA1c), which is a biomarker of the development of diabetic complications, was measured using the high-performance liquid chromatography technique.

The BMD and bone mineral content (BMC) of mouse tissues of femurs and tibiae were determined using a PIXImus mouse densitometer (GE Lunar, Waukesha, WI, USA). The BMD that was calculated from dividing BMC (mg) by the projected bone area (cm^2^) was assessed in the regions of femurs and tibiae on the 10th week. 

### 2.8. Enzyme-Linked Immunosorbent Assay (ELISA)

The plasma levels of RANKL and OPG were measured with ELISA kits, according to the manufacturer’s instructions (R&D system, Minneapolis, MN, USA). The plasma osteocalcin level was also determined using an ELISA kit (Life Technologies, Carlsbad, CA, USA). 

### 2.9. Histological Observation and Collagen Staining of Bone

The right femoral bones were fixed in 4% paraformaldehyde, and then decalcified in decalcifying solution (Sigma-Aldrich chemical, St. Louis, MO, USA) for 4–6 h. The bone tissues were dehydrated in a graded series of ethanol solutions for 18 h, followed by paraffin embedding. For histological analyses, paraffin-embedded tissues were longitudinally cut into 5 μm cryostat sections (Microm HM 520 Cryostat, GMI Inc., Ramsey, MN, USA). The tissue sections were deparaffinized and hydrated with xylene and graded ethanol. The hematoxylin and eosin (H&E) staining was applied for the histological observation of bone tissues. 

The Picrosirius red staining was employed for detecting bone collagen fibers. The tissue sections were incubated with Picrosirius red solution (Sigma-Aldrich chemical, St. Louis, MO, USA) overnight at room temperature. The images were taken using an optical Axiomager microscope system after each slide was mounted in VectaMount mounting medium (Vector Laboratories, Burlingame, CA, USA). 

### 2.10. Micro-Computed Tomography (Micro-CT)

For the in vivo analysis of bone microarchitecture, a micro-CT scanner was used to scan the mouse distal femur (VivaCT80, Scanco Medical, Brüttisellen, Switzerland). The energy was 70 kV at 114 μA/8 W intensity. Three-dimensional images were reconstructed using the reconstruction utility, and they were visualized for bone microarchitecture. For the analysis of 3D images, 1150 slices with a voxel size of 7 μm were scanned in regions from the distal femur to tibia, and 100 slices were selected.

### 2.11. Culture of MC3T3-E1 Cells 

The MC3T3-E1 cell line (ATCC CRL-2593) was obtained from the American Type Culture Collection (Manassas, VA, USA) and cultured in α-MEM that was supplemented with 10% FBS, 100 U/mL penicillin, and 100 μg/mL streptomycin at 37 °C with 5% CO_2_ in air. To differentiate MC3T3-E1 cells into osteoblast, the cells were seeded on 24-well plates at a density of 6.5 × 10^4^ cells, and they were cultured in the differentiation media of α-MEM (5.5 mM or 33 mM glucose) supplemented with 10 mM β-glycerol phosphate, 50 μg/mL ascorbic acid, and 100 nM dexamethasone for up to 21 days in the presence of 1–20 μM scopoletin. The media for cells were freshly replaced every three days. The viability of MC3T3-E1 cells that were treated with 1–20 μM scopoletin for three days was measured by using a MTT assay. 

### 2.12. Measurement of Alkaline Phosphatase (ALP) Activity and Staining

The ALP activity of MC3T3-E1 cells was performed on day 7 during differentiation. The cells were lysed in 0.5% Triton X-100, followed by incubation with 0.5 M Tris–HCl (pH 9.9) containing 6 mM *p*-nitrophenyl phosphate (*p*NP) and 1 mM MgCl_2_ at 37 °C for 2 h. The protein contents were determined, and the absorbance was read at λ = 405 nm in a microplate reader. The ALP activity was expressed as nmol *p*NP produced/min/mg protein.

The ALP staining was performed using an ALP kit (Sigma-Aldrich Chemical, St. Louis, MO, USA). After seven-day culture protocols, the cells were washed with PBS and fixed with 4% formaldehyde, rinsed with 0.05% TBS-T, and stained under protection from direct light. The ALP staining was conducted by adding naphthol/Fast Red Violet solution for 30 min. as a substrate for cells. Naphthol/Fast Red Violet solution is a mixture of Fast Red Violet (0.8 mg/mL) with 4 mg/mL Naphthol AS-BI phosphate solution in 2 M 2-amino-2-methyl-1,3-propanediol buffer (pH 9.5). The ALP staining images were photographed under light microscopy.

### 2.13. Alizarin Red S Staining

In order to measure calcium deposits, MC3T3-E1 cells were seeded on 24-well plate at density 6.5 × 10^4^ cells in differentiation media (5.5 mM or 33 mM glucose) for 21 days in the absence and presence of 1–20 μM scopoletin. The medium culture was freshly changed every three days, and Alizarin red S staining was done on day 21. The cells were rinsed in cold PBS, fixed with 70% ethanol at 4 °C for 1 h, and then stained with 40 mM Alizarin red S dye (pH 4.2) for 10 min. Calcium deposits were observed under light microscopy. 

### 2.14. Reverse Transcription Polymerase Chain Reaction (RT-PCR) Analysis

The primers (Bioneer, Daejeon, Korea) that were used to identify Runt-related transcription factor 2 (Runx2) gene were as follows: Runx2 (forward: 5′-ACATCCCCATCCATCCACTC-3′, reverse: 5′-GAAGGGTCCACTCTGGCTTT-3′, 381bp). The housekeeping gene glyceraldehyde 3-phosphate dehydrogenase (GAPDH, forward: 5′-AACTTTGGCATTGTGGAAGGG-3′, reverse: 5′-GACACATTGGGGGTAGGAACAC-3′, 224 bp) was used for an internal normalization for the coamplification with the Runx2 gene. The amplification reaction consisted of predenaturation at 95 °C for 3 min., followed by 30 cycles of denaturation at 95 °C for 35 s, annealing at 58 °C for 45 s, and elongation at 72 °C for 45 s. Final elongation was performed at 72 °C for 10 min. and posthold at 4 °C. The PCR products were run on 2% agarose gel containing 0.5 μg/mL ethidium bromide and then visualized by a TFX-20M model-UV transilluminator (Vilber Lourmat, Marne La Vallée, France).

### 2.15. Data Analysis 

The results are presented as mean ± SEM for each treatment group. Statistical analyses were performed using the Statistical Analysis Systems statistical software package (SAS Institute Inc., Cary, NC, USA). One-way ANOVA determined significance, followed by Duncan range test for multiple comparisons. Differences were considered to be significant at *p* < 0.05.

## 3. Results 

### 3.1. Effect of Scopoletin on Impairment of Osteoclastic Differentiation by Glucose

The two days-incubation with 33 mM glucose increased the viability of Raw 264.7 macrophages, which was not influenced by non-toxic scopoletin at doses of 1–20 μM (Figure 1B). In addition, 50 ng/mL RANKL *per se* enhanced the viability of Raw 264.7 macrophages in media containing 5.5 mM glucose and 33 mM glucose, which was attenuated by 1–20 μM scopoletin (Figure 1C). 

This study investigated that scopoletin boosted the differentiation impaired in high glucose-exposed osteoclasts. High glucose did not influence the ALP activity *per se* (Figure 1D). However, the stimulation with RANKL for five days reduced the TRAP activity in Raw 264.7 macrophages that were treated with 33 mM glucose (Figure 1D). In contrast, the TRAP activity dose-dependently increased in 1–20 μM scopoletin-treated macrophages that were exposed to RANKL. Consistently, the TRAP staining found that 50 ng/mL RANKL differentiated macrophages to multinucleated osteoclasts, which was hampered by high glucose (Figure 1E). However, scopoletin promoted the formation of TRAP-positive multinucleated cells. Accordingly, scopoletin invigorated the diabetes-associated impairment of osteoclast differentiation.

### 3.2. Effect of Scopoletin on Activation and Bone Resorption of Glucose-Exposed Osteoclasts

**Figure 2:**
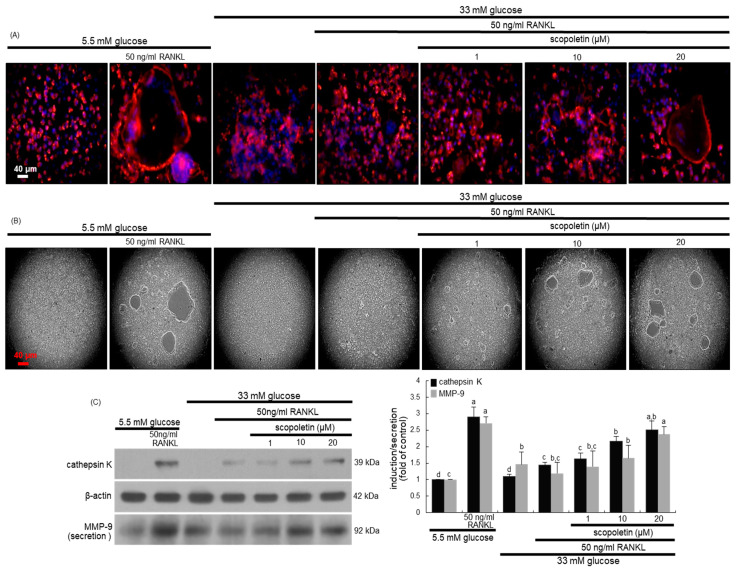
The inhibition of actin ring formation (**A**) and bone resorption (**B**), and cathepsin K induction and MMP-9 secretion (**C**) by scopoletin in glucose- and receptor activator of nuclear factor-κB-ligand (RANKL)-exposed Raw 264.7 cells. Raw 264.7 cells were cultured in α-MEM containing 5.5 mM glucose or 33 mM glucose in the absence and presence of 50 ng/mL RANKL or 1–20 μM scopoletin for five days. RANKL-differentiated cells were fixed, and rhodamine phalloidin was added to the fixed cells (**A**). Fluorescent images were taken with a fluorescence microscope. Bone resorption of Raw 264.7 cells-derived osteoclasts was assayed using a resorption assay kit (**B**). The resorbed pits on the plate were visualized under light microscopy. Original magnification of microscopic images (*n* = 6). Scale bar = 40 μm. Cell lysates and media were subject to Western blot analysis with a primary antibody against cathepsin K and MMP-9 (**C**). Representative blot data were obtained from three independent experiments, and β-actin protein was used as an internal control. The values in bar graphs (mean ± SEM, *n* = 4) not sharing a small letter are different at *p* < 0.05.

Actin ring is crucial for the formation of the actin-rich sealing zone that is responsible for optimal osteoclastic activity and bone resorption [29]. This study examined whether scopoletin restored osteoclastic activation that was damaged by high glucose. The phalloidin staining showed that actin ring formation was enhanced in RANKL-induced macrophages (Figure 2A). In addition, the bone resorption was increased in RANKL-induced osteoclasts, as evidenced by the resorbed pits on calcium-coated plates (Figure 2B). However, high glucose failed to form an actin ring structure and resorb calcium phosphate in RANKL-induced Raw 264.7 macrophages. In contrast, ≥10 μM scopoletin restored the osteoclastogenic activation by enhancing actin ring formation and bone resorption in glucose-loaded osteoclasts. Thus, scopoletin may improve the defective bone resorption in diabetic osteoclasts. 

Proteolytic enzymes of osteoclasts, such as cathepsin K and MMP-9, dissolve collagen and other matrix proteins during bone resorption [29,30]. The production of cathepsin K and MMP-9 was attenuated by loading glucose to osteoclasts (Figure 2C). The reduced induction of both matrix-degrading enzymes was reversed when scopoletin was treated with RANKL-exposed Raw 264.7 macrophages in the presence of 33 mM glucose.

### 3.3. Effects of Scopoletin on Diabetes-Associated Phenotypes of db/db Mice 

**Figure 3:**
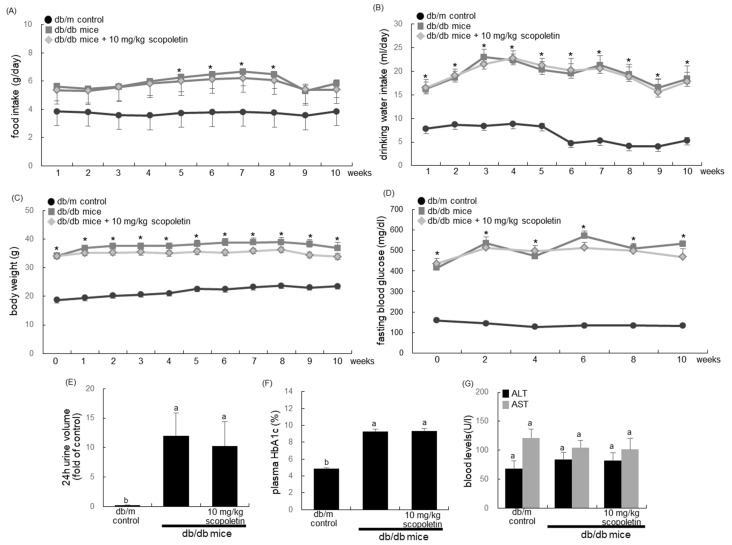
Food and drinking water intake (**A**,**B**), body weight (**C**), and fasting blood glucose level (**D**) of db/m control and db/db mice. The db/db mice were orally administrated with 10 mg/kg scopoletin daily for 10 weeks. The food intake, drinking water intake, and body weight were measured during the 10-week scopoletin supplementation, and expressed as mean ± SEM (*n* = 7). * *p* < 0.05 for db/m controls. In addition, 24-h urine volume (**E**), plasma glycated hemoglobin level (HbA1c, **F**), and plasma levels of aspartate transaminase (AST) and alanine transaminase (ALT) (**G**) were measured after the 10-week scopoletin supplementation. The values shown in bar graphs (mean ± SEM, *n* = 7) not sharing a common small letter are significantly different, at *p* < 0.05.

The food intake, drinking water intake, and body weight of db/db mice were much higher than those of db/m controls (Figure 3A–C). The increased intake of food and drinking water and increased level of fasting blood glucose were not changed when 10 mg/kg scopoletin was orally administrated to db/db mice for 10 weeks (Figure 3A,B,D). Interestingly, the body weight of db/db mice that were treated with 10 mg/kg scopoletin tended to be lower than that of untreated diabetic animals from the sixth week after its supplementation (Figure 3C). In addition, the 24-h urine volume in db/db mice was much higher (60-fold) than that in the db/m controls (Figure 3E). The treatment of scopoletin to db/db mice did not affect HbA1c and 24-h urine volume (Figure 3D,F). There was no hepatotoxicity detected in db/db mice that were treated with 10 mg/kg scopoletin. (Figure 3G). The blood levels of AST and ALT were not changed in scopoletin-administration db/db mice. Therefore, scopoletin was not effective in lowering hyperglycemia and hemoglobin glycation of db/db mice.

### 3.4. Inhibition of Bone Loss by Scopoletin in Diabetic Femur

**Figure 4:**
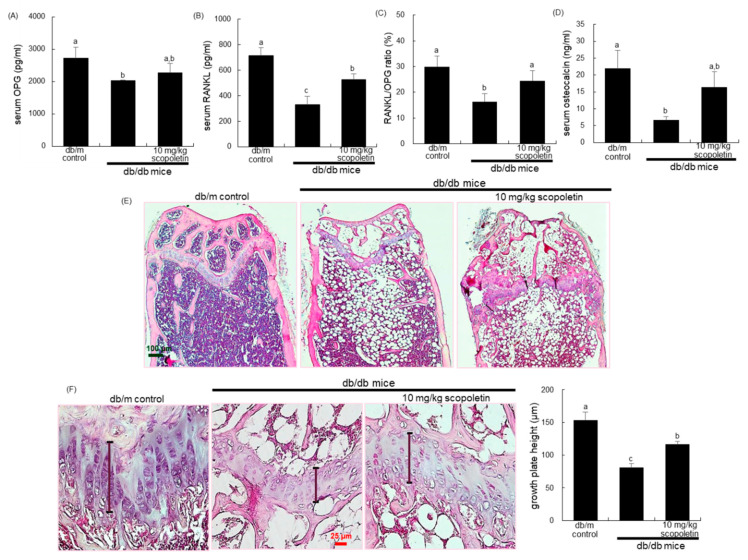
Effects of scopoletin on secretion of receptor activator of nuclear factor-κB-ligand (RANKL, **A**) and osteoprotegerin (OPG, **B**), RANKL/OPG ratio (**C**), plasma osteocalcin level (**D**), histology of trabecular bone (**E**), and epiphyseal plate thickness of growing bone (**F**). The db/db mice were orally administrated with 10 mg/kg scopoletin daily for 10 weeks. The serum levels of RANKL, OPG, and osteocalcin were determined using ELISA kits (**A**–**D**). For the observation of trabecular bone and epiphyseal plate, longitudinal sections of femoral bone tissues were H&E-stained (**E**,**F**). Images were visualized under light microscopy (four separate experiments). Scale bar = 25 and 100 μm. The values in bar graphs (mean ± SEM, *n* = 4) not sharing a small letter are different at *p* < 0.05.

OPG that is produced by osteoblasts binds to RANKL as a soluble decoy receptor, which leads to the inhibition of osteoclast differentiation and subsequent bone resorption [31,32]. The serum levels of OPG and RANKL decreased in db/db mice (Figure 4A,B). However, the scopoletin treatment elevated these levels along with an increase in the serum RANKL/OPG ratio (Figure 4C). In addition, the serum osteocalcin level was markedly diminished in db/db mice as compared with that of the db/m control mice, whereas the oral supplementation of scopoletin increased this level (Figure 4D).

**Figure 5:**
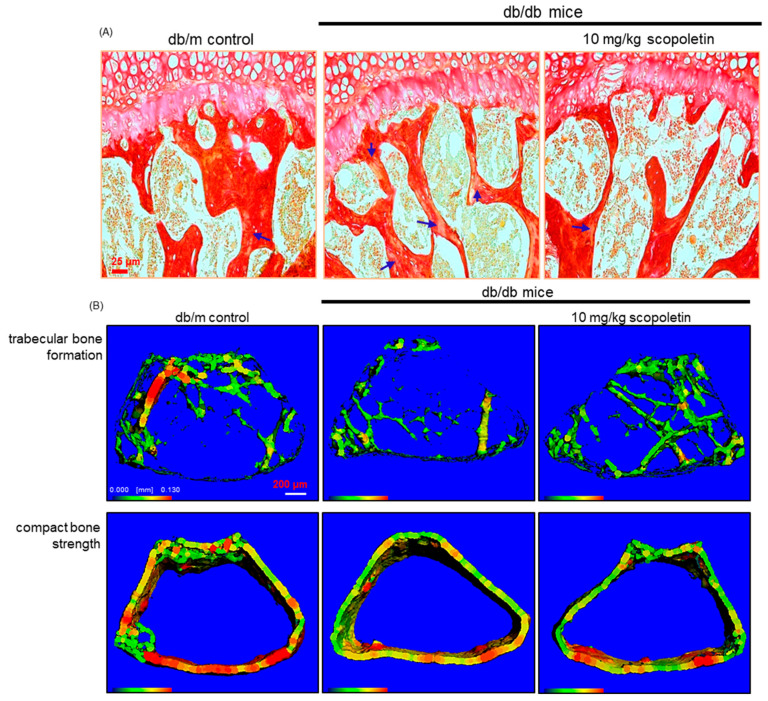
Formation of trabecular collagen fibers (**A**), and micro-computed tomographic (micro-CT) images (**B**) of femoral bone tissues in db/db mice. The db/db mice were orally administrated with 10 mg/kg scopoletin daily for 10 weeks. Picrosirius red staining was conducted for detecting trabecular bone collagen (**A**). The arrows indicate the depletion of collagen fibers in trabecular bones of the epiphysis. Representative images were visualized under light microscopy. Scale bar = 25 μm. Three-dimensional trabecular bone and compact bone are shown by micro-CT reconstruction at the distal femurs (**B**). For the 3D images analysis, 1150 slices with a voxel size of 7 μm were scanned in regions from the distal femur to tibia, and 100 slices were selected. Scale bar = 200 μm.

The H&E staining showed that the formation of trabecular bones of epiphysis and metaphysis was diminished in diabetic femurs with the appearance of osteoporotic bone loss in the metaphysis, trabecular bone spacing, and thin epiphyseal plate (Figure 4E,F). When db/db mice were supplemented with 10 mg/kg scopoletin, more trabecular bones were formed in femoral epiphysis with the epiphyseal plate being thicker, in contrast with those of the db/db mice. On the other hand, there were whitish patchy marks (arrows) observed in femoral trabecular bone tissues of db/db mice, as evidenced by picrosirius red staining (Figure 5A). In marked contrast, the collagen fiber staining in scopoletin-treated db/db mice was indistinguishable from that of the db/m control animals (Figure 5A). Accordingly, it can be assumed that scopoletin restored collagen formation was reduced in hyperglycemia-induced mice. 

This study found that the BMD and BMC of femoral and tibial bones declined in db/db mice (Table 2). The BMD and BMC were significantly enhanced when 10 mg/kg scopoletin was challenged with db/db mice. In addition, the trabecular bone mass and cortical bone thickness decreased in the femurs of diabetic mice, as evidenced by 3D micro-CT images (Figure 5B). The trabecular bones were much denser in db/db mice that were treated with scopoletin, which indicated that its administration protected mice from diabetic bone loss. 

### 3.5. Improvement of Bone Mineralization of Glucose-Exposed Osteoblasts by Scopoletin

**Figure 6:**
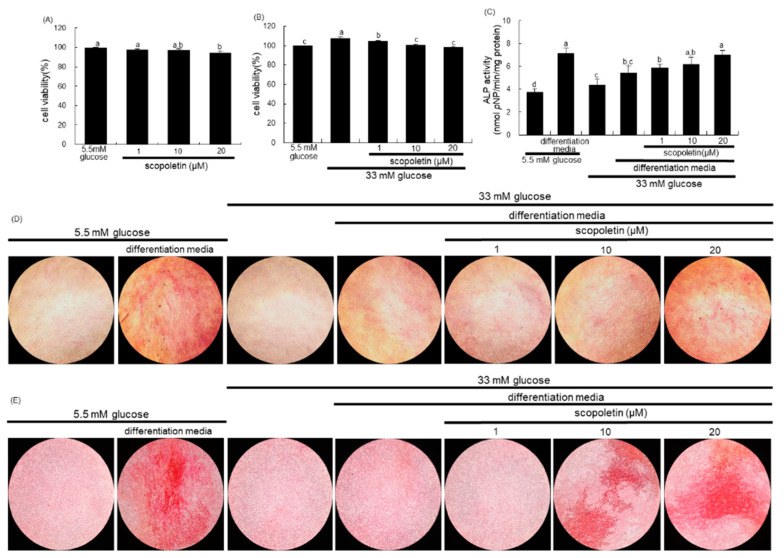
Cell toxicity of scopoletin (**A**,**B**), and elevation of alkaline phosphatase (ALP) activity (**C**,**D**) and calcium deposit (**E**) in pre-osteoblastic MC3T3-E1 cells treated with scopoletin. MC3T3-E1 cells were cultured in α-MEM-differentiation media containing 5.5 mM glucose or 33 mM glucose for three days in the absence and presence of 1–20 μM scopoletin. Cell viability was measured by the MTT assay and expressed as percent cell survival relative to untreated glucose controls (**A**,**B**, cell viability = 100%, mean ± SEM, *n* = 4). For the measurement of ALP activity (**C**), the MC3T3-E1 cells were cultured in α-MEM-differentiation media containing 5.5 mM glucose or 33 mM glucose in the presence of 1–20 μM scopoletin for seven days. The ALP enzyme activity in media was expressed as nmol *p*-nitrophenyl phosphate (*p*NP)/min/mg protein. Absorbance was measured at λ = 450 nm and compared with *p*-nitrophenol standard (mean ± SEM, *n* = 3). Values in bar graphs not sharing a small letter are different at *p* < 0.05. The ALP staining was visualized under light microscopy (**D**). The calcium deposit (bone nodule formation) was measured by Alizarin red S staining (**E**). MC3T3-E1 cells were cultured in α-MEM-differentiation media containing 5.5 mM glucose or 33 mM glucose in the presence of 1–20 μM scopoletin for 21 days. Microphotographs were representative of 21 day-grown osteoblasts on the well slides. The calcium deposits were visualized under light microscopy. Magnification: 100-fold.

There was no toxicity of MC3T3-E1 cells observed in response to the treatment of 1–20 µM scopoletin for three days (Figure 6A). Non-toxic scopoletin corrected the increased proliferation of MC3T3-E1 cells due to 33 mM glucose (Figure 6B). ALP is crucial for bone mineralization as a biochemical marker that is useful at the early and mid-stage of bone formation [33]. The colorimetric enzyme assay revealed that the ALP activity was markedly enhanced in MC3T3-E1 cells that were cultured in osteogenic differentiation media (Figure 6C). High glucose suppressed the ALP activity of MC3T3-E1 cells, while treatment with 1–20 µM scopoletin enhanced this activity in diabetic MC3T3-E1 cells (Figure 6C). The differentiated MC3T3-E1 cells consistently displayed strong ALP staining, which was attenuated in the presence of high glucose (Figure 6D). However, the treatment of scopoletin to diabetic MC3T3-E1 osteoblasts enhanced the ALP levels in a dose-dependent manner. 

Alizarin red S staining was introduced to visualize bone nodule formation and calcium deposits in osteoblasts. The Alizarin red S staining revealed no calcium deposit in undifferentiated MC3T3-E1 cells, whereas a strong staining of Alizarin red S was detected in cells that were cultured in osteogenic differentiation media for 21 days (Figure 6E). In contrast, calcium accumulation and mineralized bone nodule formation were highly reduced in diabetic MC3T3-E1 cells that were cultured in osteogenic differentiation media. Calcium deposits were significantly enhanced when ≥10 µM scopoletin was administered to diabetic MC3T3-E1 cells in osteogenic media. Taken together, scopoletin may boost the differentiation and bone mineralization of diabetic osteoblasts. 

### 3.6. Scopoletin Induction of Osteogenic Proteins Quiescent Due to Glucose

**Figure 7:**
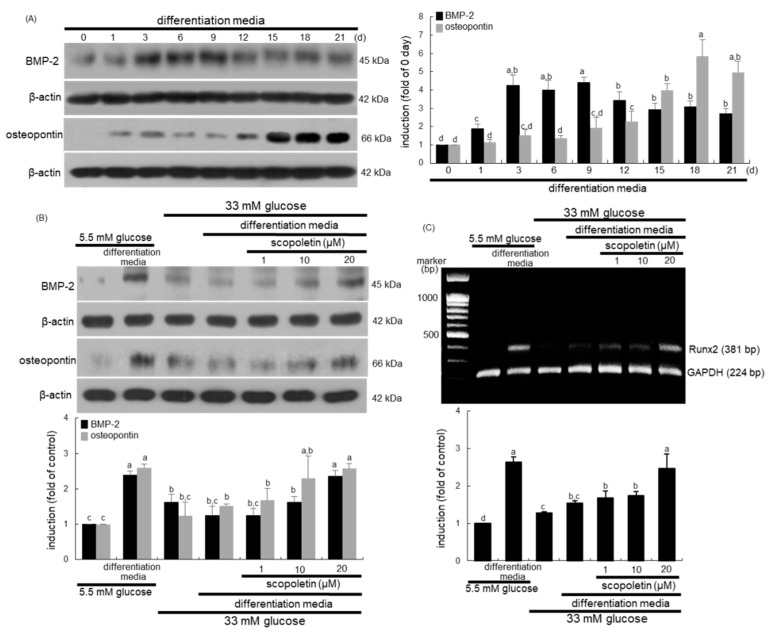
The temporal responses of induction of bone morphogenetic protein (BMP)-2 and osteopontin (**A**) and effects of scopoletin on induction of BMP-2 and osteopontin (**B**) and on Runt-related transcription factor 2 (Runx2) transcription (**C**) in pre-osteoblastic MC3T3-E1 cells. MC3T3-E1 cells were cultured in α-MEM-differentiation media for up to 21 days. Cell lysates were subject to Western blot analysis with a primary antibody against BMP-2 and osteopontin (**A**,**B**). β-Actin protein was used as an internal control. Values in bar graphs (mean ± SEM, *n* = 3) not sharing a small letter are different at *p* < 0.05. The transcription of Runx2 was measured by Reverse transcription polymerase chain reaction assay, and the glyceraldehyde 3-phosphate dehydrogenase (GAPDH) gene was used for the internal controls (**C**). Respective values (mean ± SEM, *n* = 3) not sharing a small letter are different at *p* < 0.05.

This study investigated whether scopoletin ameliorated the expression of osteogenic proteins of BMP-2, osteopontin, and Runx2 during 21 day-osteoblastic differentiation failed by glucose. Osteopontin is one of the abundant non-collagenous proteins in the bone matrix [34]. The cellular BMP-2 expression temporally peaked at the early–mid stage of differentiation (Figure 7A). On the contrary, the cellular osteopontin expression highly increased at the late stage of differentiation. The BMP-2 induction was markedly diminished when MC3T3-E1 cells were exposed to 33 mM glucose-containing differentiation media for three days (Figure 7B). Additionally, the osteopontin expression of glucose-loaded MC3T3-E1 cells was reduced during 15 day-differentiation (Figure 7B). The treatment of pre-osteoblastic cells with 1–20 µM scopoletin in 33 mM glucose-containing osteogenic media dose-dependently stimulated the induction of both BMP-2 and osteopontin (Figure 7B). 

Runx2 induces the osteoblast phenotype via BMP signaling [35], and its transcription of MC3T3-E1 cells enhances at the mid-stage of the 21 day-differentiation period [36]. The RT-PCR data revealed that the transcriptional Runx2 level was highly enhanced in osteoblasts, which was dampened by high glucose (Figure 7C). However, the treatment with 20 μM scopoletin markedly stimulated Runx2 transcription in glucose-loaded osteoblasts (Figure 7C). 

## 4. Discussion

**Figure 8:**
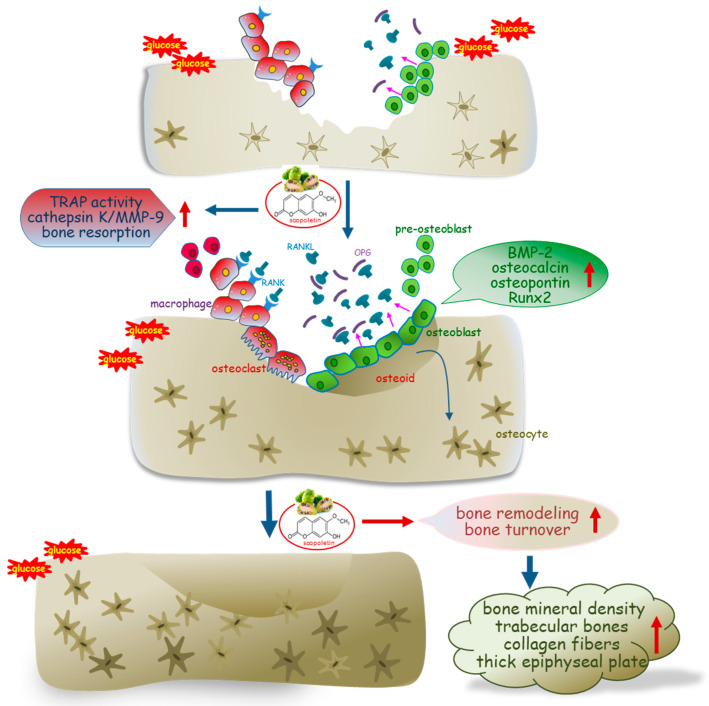
Schematic diagram showing the effects of scopoletin on bone turnover and remodeling in type 2 diabetic mouse model. The arrows indicate activation by scopoletin. TARP, tartrate-resistant acid phosphatase; RANK, receptor activator of nuclear factor-κB; RANKL, RANK ligand; OPG, osteoprotegerin; BMP-2, bone morphogenetic protein-2; Runx2, Runt-related transcription factor 2.

Eight main findings can be derived from this study. (1) Submicromolar scopoletin stimulated the formation of TRAP-positive multinucleated cells that are impaired by glucose. (2) The glucose loading inhibited the formation of actin ring structure, bone resorption, and induction of matrix-degrading enzymes of cathepsin K and MMP-9 in osteoclasts, which was reversed by culturing 1–20 μM scopoletin. (3) The oral administration of 10 mg/kg scopoletin did not display beneficial effects on diabetes-associated hyperglycemic phenotypes in diabetic animals, despite having no hepatotoxicity. (4) Scopoletin elevated serum RANKL/OPG ratio and osteocalcin level reduced in db/db mice. (5) When db/db mice were supplemented with scopoletin, the formation of trabecular bones and accumulation of collagen fibers were elevated in femoral epiphysis and metaphysis with thicker epiphyseal plate. (6) The administration of scopoletin to diabetic mice enhanced cortical bone thickness with an increase in BMD and BMC. (7) Culturing diabetic MC3T3-E1 osteoblasts with scopoletin enhanced ALP activity and deposits of mineralized bone nodules. (8) The treatment of diabetic osteoblasts with scopoletin stimulated the cellular induction of both BMP-2 and osteopontin and Runx2 transcription. Accordingly, the administration of scopoletin protected diabetic mice from bone loss through enhancing bone turnover of bone-degrading osteoclasts and bone-forming osteoblasts via the RANKL-OPG system and BMP-2-Runx2 signaling (Figure 8).

An increased risk of bone fractures is reported in individuals with type 2 diabetes in spite of normal, or even increased, BMD [3,5,8]. Thus, it can be assumed that patients with type 2 diabetes may have abnormalities in bone microarchitecture and/or material composition that are key determinants of bone quality [4]. Individuals with type 1 diabetes, i.e., insulin-dependent diabetes, have considerably low BMD alongside reduced bone mineralization and suppressed bone turnover, which leads to enhancing vertebral and hip fractures [9]. Accordingly, the insulin deficiency in individuals with type 1 diabetes may cause detrimental effects on bone density and bone strength. Several studies show that insulin can preserve robust bone density and bone strength, presumably through direct and/or indirect effects on anabolic bone formation [10,20,37]. However, the insulin application is associated with an increased risk of osteoporosis and brittle fractures in patients with type 2 diabetes [13,14]. Thus, better knowledge regarding how the use of insulin as an anabolic agent influences skeletal tissues greatly needed in view of patients with diabetes. Recently, much attention has been focused on the negative or positive impact of anti-diabetic drugs on bone turnover [11,38,39]. Thiazolidinediones increase the bone loss and risk of fractures, and hamper osteoblastogenesis via decreasing the Runx2 transcription factor, insulin-like growth factor-1, and Wnt signaling pathways [6,12,39]. Metformin has a neutral or positive effect on bone health and reduces the risk of fractures [39,40]. Sodium-glucose co-transporter 2 inhibitors may cause bone loss or increase the risk of fractures due to alterations in the bone microarchitecture [11,39]. Safety concerns of anti-diabetic drugs are crucial in the management of diabetes because the pathophysiological mechanisms accentuating the risk of fractures in individuals with type 2 diabetes are complicated. 

Bone turnover is reduced in type 2 diabetes, as compared with non-diabetic controls, which implies an adverse effect of hyperglycemia on bone turnover [9,18]. There is growing evidence that an abnormal state between bone resorption and formation reduces bone turnover in type 2 diabetes, despite conflicting reports [4]. This study showed that the RANKL-induced differentiation and bone resorption were reduced in glucose-loaded osteoclasts. In addition, the osteoblastic differentiation and mineralization were inhibited in diabetic osteoblasts through disturbing signal transduction and transcriptional regulation of BMP-2 and Runx2, which suggests the suppression of bone formation. These results revealed that hyperglycemia inhibited the optimal bone remodeling of osteoclasts and osteoblasts, assumedly leading to the occurrence of bone loss and fracture. The db/db mice are currently used as a mouse model of type 2 diabetes with a mutation in the gene encoding the leptin receptor, and they are susceptible to obesity and insulin resistance due to the deficiency of leptin [41]. The current study found that BMD and BMC decreased in obese db/db mice with a concurrent reduction of bone turnover markers of osteocalcin, RANKL, and OPG. Furthermore, the formation of trabecular bones and the accumulation of collagen fibers were diminished in diabetic mice with thin epiphyseal plate, which, in turn, might lead to more fragile bone. The reduction of markers of both bone formation and bone resorption reflects a state of low bone turnover in patients with diabetes [42]. One study shows that the suppression of bone turnover is independent of BMD in patients with type 2 diabetes with a higher risk of fracture [19]. Additionally, the impairment in bone strength in type 2 diabetes is due to impaired bone quality that is assumedly caused by elevated AGE accumulation and low bone turnover [20].

Currently, most glucose-lowering medications may have a neutral effect on fracture risk, but caution should be taken with the bone quality of diabetic individuals at a higher risk of fractures. Thus, the management of fracture risk in diabetic patients remains challenging, and alternative and effective therapies with less harmful side effects are required. Natural compounds that suppress osteoclast commitment may have therapeutic value in treating diabetic pathologies that are associated with bone resorption. The hydroxycoumarin scopoletin, as a potential anti-diabetic agent, ameliorates insulin resistance [26,27]. The present study found that scopoletin ameliorated osteoclastogenesis by RANKL in diabetic osteoclasts, in parallel with robust osteobalstogenesis in diabetic osteoblasts via the induction of BMP-2 and Runx2. Furthermore, scopoletin enhanced bone density, although it was not effective in the glucose-lowering strategy. Several studies show that individuals with poor glycemic control are at a higher risk of fracture when compared with those with lower HbA1c levels [43,44]. Scopoletin did not lower the blood levels of glucose and HbA1c, which was indicative of poor glycemic control. Nevertheless, scopoletin boosted the formation of trabecular bones and collagen fibers in femoral epiphysis and metaphysis of type 2-diabetic mice with a thicker epiphyseal plate. There are few studies dealing with natural anti-diabetic agents that improve bone remodeling and turnover in diabetic episodes related to bone fracture and loss. Our in vitro study showed that coumarin accelerated the differentiation and activation in both diabetic osteoclasts and osteoblasts [28]. The *p*-hydroxycinnamic acid intake inhibits bone loss in femoral diaphyseal and metaphyseal tissues of streptozotocin-induced diabetic rats [45]. Oral supplementation with *p*-coumaric acid suppresses the spontaneous destruction of periodontal tissue in streptozotocin-treated mice through enhancing the anti-inflammatory, anti-osteoclastogenic, and antioxidant defense systems [46]. This natural compound enhanced the plasma levels of bone markers of osteocalcin, RANKL, and OPG in db/db mice, presumably enhancing the diabetes-associated impairment of bone turnover and remodeling. However, additional studies are required to determine the optimal management strategies of scopoletin reversing decreased BMD and an increased risk of bone fracture in diabetes. In addition, the absence of consistent human studies dealing with dietary scopoletin is the limitation of this study.

## 5. Conclusions

The current study demonstrated that the oral administration of scopoletin inhibited the loss of trabecular bones and collagen fibers in the femurs of diabetic mice. Scopoletin promoted osteoclastogenic differentiation and activation of RANKL-challenged diabetic Raw 264.7 osteoclasts. Concurrently, scopoletin enhanced osteblastogenic differentiation with an increase in ALP activity and calcium deposit in glucose-loaded MC3T3-E1 osteoblasts. The aberrant impairment of osteoclastogenic born resorption was optimally improved by scopoletin via the stabilization of the RANKL/OPG system of diabetic osteoclasts. The BMP-2-Runx2 signaling pathway was responsible for disrupting osteoblastogenic differentiation under glucotoxic condition. Scopoletin ameliorated bone turnover and remodeling in diabetic mice along with an increase in BMD through a well-functioning RANKL/OPG system and BMP-2-Runx2 signaling pathway. Therefore, scopoletin can be a substitute for the detrimental side effects of conventional therapies in diabetic osteopathy. 

## Figures and Tables

**Table 1:** Organ weights in db/m control and db/db mice.

Group	Liver	Kidney	Spleen	Pancreas	Heart
db/m control	1.426 ± 0.038 ^b^	0.384 ± 0.011 ^b^	0.087 ± 0.009 ^a^	0.276 ± 0.027 ^a^	0.139 ± 0.009 ^a^
db/db mice	2.241 ± 0.127 ^a^	0.452 ± 0.020 ^a^	0.037 ± 0.006 ^b^	0.249 ± 0.030 ^a^	0.136 ± 0.012 ^a^
db/db mice + 10 mg/kg scopoletin	2.203 ± 0.136 ^a^	0.423 ± 0.019 ^a,b^	0.042 ± 0.006 ^b^	0.228 ± 0.026 ^a^	0.128 ± 0.003 ^a^

The db/db mice were supplemented with 10 mg/kg scopoletin daily for 10 weeks. The organ weights (g) were measured after the 10-week scopoletin supplementation. Respective values in same column (mean ± SEM, *n* = 7) not sharing a small letter are different at *p* < 0.05.

**Table 2:** Osteogenic activity of scopoletin in db/db mice.

Bones	Parameters	db/m Control	db/db Mice	db/db Mice + 10 mg/kg Scopoletin
femurs	BMD (mg/cm)	63.80 ± 1.30 ^a^	48.31 ± 1.38 ^c^	55.27 ± 1.02 ^b^
	BMC (mg)	7.86 ± 0.26 ^a^	5.71 ± 0.29 ^c^	6.43 ± 1.20 ^b^
	Area (cm)	0.12 ± 0.002 ^a^	0.12 ± 0.001 ^a^	0.12 ± 0.002 ^a^
tibiae	BMD (mg/cm)	50.01 ± 0.82 ^a^	36.44 ± 0.76 ^c^	38.79 ± 0.67 ^b^
	BMC (mg)	4.86 ± 0.26 ^a^	3.86 ± 0.14 ^c^	4.00 ± 0.00 ^b^
	Area (cm)	0.10 ± 0.003 ^a^	0.10 ± 0.000 ^a^	0.10 ± 0.002 ^a^

The db/db mice were supplemented with 10 mg/kg scopoletin daily for 10 weeks. The bone mineral density (BMD) and bone mineral content (BMC) of mouse tissues of femurs and tibiae were determined using a PIXImus mouse densitometer. Respective values in same row (mean ± SEM, *n* = 7) not sharing a small letter are different at *p* < 0.05.

## Data Availability

All the data presented in this study are included in the article.

## Abbreviations

AGE: advanced glycation end products; ALP, alkaline phosphatase; BMC, bone mineral concentration; BMD, bone mineral density; BMP-2, bone morphogenetic protein; MMP-9, matrix metalloproteinase-9; MTT, 3-(4, 5-dimetylthiazol-yl)-diphenyl tetrazolium bromide; OPG, osteoprotegerin; RANKL, receptor activator of nuclear factor-κΒ ligand; Runx2, Runt-related transcription factor 2; TRAP, tartrate-resistant acid phosphatase.

## References

[1] Kurra S., Siris E. (2011). Diabetes and bone health: The relationship between diabetes and osteoporosis-associated fractures. Diabetes Metab. Res. Rev..

[2] Schwartz A.V., Sellmeyer D.E. (2007). Diabetes, fracture, and bone fragility. Curr. Osteoporos. Rep..

[3] Janghorbani M., van Dam R.M., Willett W.C., Hu F.B. (2007). Systematic review of type 1 and type 2 diabetes mellitus and risk of fracture. Am. J. Epidemiol..

[4] Farr J.N., Khosla S. (2016). Determinants of bone strength and quality in diabetes mellitus in humans. Bone.

[5] Vestergaard P. (2007). Discrepancies in bone mineral density and fracture risk in patients with type 1 and type 2 diabetes-a meta-analysis. Osteoporos. Int..

[6] Khazai N.B., Beck G.R., Umpierrez G.E. (2009). Diabetes and fractures: An overshadowed association. Curr. Opin. Endocrinol. Diabetes Obes..

[7] Nyman J.S., Even J.L., Jo C.H., Herbert E.G., Murry M.R., Cockrell G.E., Wahl E.C., Bunn R.C., Lumpkin C.K., Fowlkes J.L. (2011). Increasing duration of type 1 diabetes perturbs the strength-structure relationship and increases brittleness of bone. Bone.

[8] Shanbhogue V.V., Mitchell D.M., Rosen C.J., Bouxsein M.L. (2016). Type 2 diabetes and the skeleton: New insights into sweet bones. Lancet Diabetes Endocrinol..

[9] Costantini S., Conte C. (2019). Bone health in diabetes and prediabetes. World J. Diabetes.

[10] Thrailkill K.M., Lumpkin C.K., Bunn R.C., Kemp S.F., Fowlkes J.L. (2005). Is insulin an anabolic agent in bone? Dissecting the diabetic bone for clues. Am. J. Physiol. Endocrinol. Metab..

[11] Ye Y., Zhao C., Liang J., Yang Y., Yu M., Qu X. (2019). Effect of sodium-glucose co-transporter 2 inhibitors on bone metabolism and fracture risk. Front. Pharmacol..

[12] Horii T., Iwasawa M., Kabeya Y., Shimizu J., Atsuda K. (2019). Investigating the risk of bone fractures in elderly patients with type 2 diabetes mellitus: A retrospective study. BMC Endocr. Disord..

[13] Losada-Grande E., Hawley S., Soldevila B., Martinez-Laguna D., Nogues X., Diez-Perez A., Puig-Domingo M., Mauricio D., Prieto-Alhambra D. (2017). Insulin use and excess fracture risk in patients with type 2 diabetes: A propensity-matched cohort analysis. Sci. Rep..

[14] Zhang Y., Chen Q., Liang Y., Dong Y., Mo X., Zhang L., Zhang B. (2019). Insulin use and fracture risk in patients with type 2 diabetes: A meta-analysis of 138,690 patients. Exp. Ther. Med..

[15] Al-Hariri M. (2016). Sweet bones: The pathogenesis of bone alteration in diabetes. J. Diabetes Res..

[16] Yamamoto M., Sugimoto T. (2016). Advanced glycation end products, diabetes, and bone strength. Curr. Osteoporos. Rep..

[17] Yamagishi S.I. (2011). Role of advanced glycation end products (AGEs) in osteoporosis in diabetes. Curr. Drug Targets.

[18] Starup-Linde J. (2013). Diabetes, biochemical markers of bone turnover, diabetes control, and bone. Front. Endocrinol..

[19] Dobnig H., Piswanger-Sölkner J.C., Roth M., Obermayer-Pietsch B., Tiran A., Strele A., Maier E., Maritschnegg P., Sieberer C., Fahrleitner-Pammer A. (2006). Type 2 diabetes mellitus in nursing home patients: Effects on bone turnover, bone mass, and fracture risk. J. Clin. Endocrinol. Metab..

[20] Napoli N., Chandran M., Pierroz D.D., Abrahamsen B., Schwartz A.V., Ferrari S.L. (2017). Mechanisms of diabetes mellitus-induced bone fragility. Nat. Rev. Endocrinol..

[21] Ding Z., Dai Y., Hao H., Pan R., Yao X., Wang Z. (2008). Anti-inflammatory effects of scopoletin and underlying mechanisms. Pharmaceut. Biol..

[22] Ojewole J.A., Adesina S.K. (1983). Mechanism of the hypotensive effect of scopoletin isolated from the fruit of Tetrapleura tetraptera. Planta Med..

[23] Chang W.S., Chang Y.H., Lu F.J., Chiang H.C. (1994). Inhibitory effects of phenolics on xanthine oxidase. Anticancer Res..

[24] Shaw C.Y., Chen C.H., Hsu C.C., Chen C.C., Tsai Y.C. (2003). Antioxidant properties of scopoletin isolated from Sinomonium acutum. Phytother. Res..

[25] Shalan N.A., Mustapha N.M., Mohamed S. (2017). Noni leaf and black tea enhance bone regeneration in estrogen-deficient rats. Nutrition.

[26] Kalpana K., Priya C.S., Dipti N., Vidhya R., Anuradha C.V. (2019). Supplementation of scopoletin improves insulin sensitivity by attenuating the derangements of insulin signaling through AMPK. Mol. Cell. Biochem..

[27] Chang W.C., Wu S.C., Xu K.D., Liao B.C., Wu J.F., Cheng A.S. (2015). Scopoletin protects against methylglyoxal-induced hyperglycemia and insulin resistance mediated by suppression of advanced glycation endproducts (AGEs) generation and anti-glycation. Molecules.

[28] Lee E.J., Kang M.K., Kim Y.H., Kim D.Y., Oh H., Kim S.I., Oh S.Y., Na W., Kang Y.H. (2020). Coumarin ameliorates impaired bone turnover by inhibiting the formation of advanced glycation end products in diabetic osteoblasts and osteoclasts. Biomolecules.

[29] Teitelbaum S.L. (2011). The osteoclast and its unique cytoskeleton. Ann. N. Y. Acad. Sci..

[30] Charles J.F., Aliprantis A.O. (2014). Osteoclasts: More than bone eaters. Trends Mol. Med..

[31] Schneeweis L.A., Willard D., Milla M.E. (2005). Functional dissection of osteoprotegerin and its interaction with receptor activator of NF-κB ligand. J. Biol. Chem..

[32] Hofbauer L.C., Schoppet M. (2004). Clinical implications of the osteoprotegerin/RANKL/RANK system for bone and vascular diseases. JAMA.

[33] Rutkovskiy A., Stensløkken K.O., Vaage I.J. (2016). Osteoblast differentiation at a glance. Med. Sci. Monit. Basic Res..

[34] Jimi E., Hirata S., Shin M., Yamazaki M., Fukushima H. (2010). Molecular mechanisms of BMP-induced bone formation: Cross-talk between BMP and NF-κB signaling pathways in osteoblastogenesis. Jpn. Dent. Sci. Rev..

[35] Komori T. (2010). Regulation of osteoblast differentiation by Runx2. Adv. Exp. Med. Biol..

[36] Antika L.D., Lee E.J., Kim Y.H., Kang M.K., Park S.H., Kim D.Y., Oh H., Choi Y.J., Kang Y.H. (2017). Dietary phlorizin enhances osteoblastogenic bone formation through enhancing β-catenin activity via GSK-3β inhibition in a model of senile osteoporosis. J. Nutr. Biochem..

[37] Yan W., Li X. (2013). Impact of diabetes and its treatments on skeletal diseases. Front. Med..

[38] Stage T.B., Christensen M.M.H., Jørgensen N.R., Beck-Nielsen H., Brøsen K., Gram J., Frost M. (2018). Effects of metformin, rosiglitazone and insulin on bone metabolism in patients with type 2 diabetes. Bone.

[39] Adil M., Khan R.A., Kalam A., Venkata S.K., Kandhare A.D., Ghosh P., Sharma M. (2017). Effect of anti-diabetic drugs on bone metabolism: Evidence from preclinical and clinical studies. Pharmacol. Rep..

[40] Bahrambeigi S., Yousefi B., Rahimi M., Shafiei-Irannejad V. (2019). Metformin; an old antidiabetic drug with new potentials in bone disorders. Biomed. Pharmacother..

[41] Alpers C.E., Hudkins K.L. (2011). Mouse models of diabetic nephropathy. Curr. Opin. Nephrol. Hypertens..

[42] Hygum K., Starup-Linde J., Harsløf T., Vestergaard P., Langdahl B.L. (2017). Diabetes mellitus, a state of low bone turnover-a systematic review and meta-analysis. Eur. J. Endocrinol..

[43] Conway B.N., Long D.M., Figaro M.K., May M.E. (2016). Glycemic control and fracture risk in elderly patients with diabetes. Diabetes Res. Clin. Pract..

[44] Vavanikunnel J., Charlier S., Becker C., Schneider C., Jick S.S., Meier C.R., Meier C. (2019). Association between glycemic control and risk of fracture in diabetic patients: A nested case-control study. J. Clin. Endocrinol. Metab..

[45] Yamaguchi M., Uchiyama S., Lai Y.L. (2007). Oral administration of phytocomponent *p*-hydroxycinnamic acid has a preventive effect on bone loss in streptozotocin-induced diabetic rats. Int. J. Mol. Med..

[46] Bhattarai G., Min C.K., Jeon Y.M., Bashyal R., Poudel S.B., Kook S.H., Lee J.C. (2019). Oral supplementation with p-coumaric acid protects mice against diabetes-associated spontaneous destruction of periodontal tissue. J. Periodontal Res..

